# The biology and dynamics of mammalian cortical granules

**DOI:** 10.1186/1477-7827-9-149

**Published:** 2011-11-17

**Authors:** Min Liu

**Affiliations:** 1 Department of Life Science and Graduate Institute of Biotechnology, Private Chinese Culture University, Taipei, Republic of China

**Keywords:** mammalian cortical granules, oocytes, fertilization, exocytosis, SNARE proteins, calcium, cortical granule free domains, pre-fertilization release, heterogeneity

## Abstract

Cortical granules are membrane bound organelles located in the cortex of unfertilized oocytes. Following fertilization, cortical granules undergo exocytosis to release their contents into the perivitelline space. This secretory process, which is calcium dependent and SNARE protein-mediated pathway, is known as the cortical reaction. After exocytosis, the released cortical granule proteins are responsible for blocking polyspermy by modifying the oocytes' extracellular matrices, such as the zona pellucida in mammals. Mammalian cortical granules range in size from 0.2 um to 0.6 um in diameter and different from most other regulatory secretory organelles in that they are not renewed once released. These granules are only synthesized in female germ cells and transform an egg upon sperm entry; therefore, this unique cellular structure has inherent interest for our understanding of the biology of fertilization. Cortical granules are long thought to be static and awaiting in the cortex of unfertilized oocytes to be stimulated undergoing exocytosis upon gamete fusion. Not till recently, the dynamic nature of cortical granules is appreciated and understood. The latest studies of mammalian cortical granules document that this organelle is not only biochemically heterogeneous, but also displays complex distribution during oocyte development. Interestingly, some cortical granules undergo exocytosis prior to fertilization; and a number of granule components function beyond the time of fertilization in regulating embryonic cleavage and preimplantation development, demonstrating their functional significance in fertilization as well as early embryonic development. The following review will present studies that investigate the biology of cortical granules and will also discuss new findings that uncover the dynamic aspect of this organelle in mammals.

## Background

**Figure 1:**
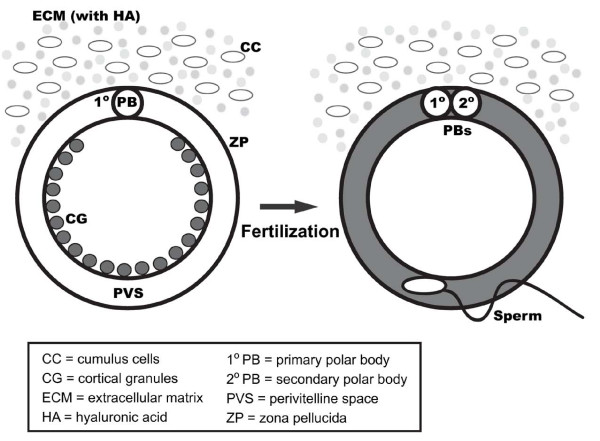
**Mammalian cortical granule exocytosis during fertilization**. Cortical granules are secretory vesicles located in the cortex of unfertilized oocytes (shown in circles in grey). Upon gamete fusion, cortical granules undergo exocytosis and release their contents into the extracellular space to establish a block of polyspermy. For clarity purpose, cellular structures are not illustrated in scale. CC = cumulus cells; CG = cortical granules; ECM = extracellular matrix; HA = hyaluronic acid; 1^o^PB = primary polar body; 2^o^PB = secondary polar body; PVS = perivitelline space; ZP = zona pellucid.

Mammalian fertilization is a sequence of coordinated events involving multiple steps of mutual recognitions between haploid male and female gametes. In mammals, fertilizable oocytes are ovulated along with the first polar body, as well as the extracellular matrix (ECM) zona pellucid (ZP) which is composed of three glycoproteins, ZP1, ZP2, and ZP3 [1,2] and the cumulus oophorus which is made of several layers of ovarian follicular granulose cells embedded in hyaluronic acid-containing ECM (Figure 1). Passage of a sperm through these semi-permeable investments surrounding an oocyte is absolutely essential in the initiation of fertilization. First, sperm need to pass through the cumulus oophorus and they do so by disrupting the macromolecular structure of the matrix [3] and by sperm hyaluronidase activity [4]. Next, formation of a pathway through the ZP is triggered by the recognition of a cognate receptor on sperm and the ZP3 glycoprotein of the oocyte by protein-carbohydrate recognition [5,6], followed by the induction of proteolytic enzyme release from the sperm head termed acrosome reaction. Sperm penetration through the ZP is subsequently facilitated by the binding between acrosome reacted sperm and ZP2 glycoproteins [7]. Most studies of mammalian fertilization support the model described above regarding gamete recognition and interaction; however, a recent report has revealed that ZP3 glycoprotein is not essential and sufficient to induce sperm acrosome reaction while binding to ZP2 is account for sperm-egg recognition during fertilization in mice [8]. Whether this model for gamete recognition is adequate for all mammals still needs to be determined experimentally.

Prior to fertilization, a sperm needs to swim through the perivitelline space (PVS) and fuses with the oocyte through a fertilin/integrin- mediated adhesion process [9-15]. Following gamete fusion, the fertilized oocyte resumes meiosis and protrudes the second polar body, which marks the end of fertilization process. However, a successful fertilization is not guaranteed unless preventative mechanism(s) inhibiting fertilization by more than one sperm, a condition known as polyspermy, is established immediately following gamete fusion since polyploidy is fatal to an embryo. Physiological polyspermy or penetration of the oocyte by more than a single sperm occurs in numerous species including insects, reptiles, and birds, while mammalian polyspermic fertilization is considered abnormal, which leads to developmental failure of the zygote and spontaneous abortion in humans. A recent study of porcine oocytes reveals that pig is a species with a high incidence of polyspermy under physiological conditions or *in vitro *fertilization [16]. Interestingly, porcine oocyte cytoplasm has the capability to remove the accessory sperm [17] and some poly-pronuclear pig eggs can develop to term if accessory sperm do not interrupt the embryo genome [18], demonstrating that this species depends on unique mechanisms to deal with the occurrence of polyspermy. Nevertheless, a defense against polyspermic fertilization needs to be established rapidly after fertilization under most circumstances in mammals.

In mammals, fertilization occurs internally (e.g. cervix, uterotubal junction) and female reproductive tract limits the arrival of sperm to the fertilization site [19]. In mice, 50 million sperms are ejaculated and only 100 to 200 sperm actually reach the site of ovulated oocytes [20], supporting by the observation that *in vitro *fertilization has higher incidence of polyspermy in animals and humans [16,21,22] and the frequency of polyspermic fertilization is directly related to the sperm concentration [23]. However, this mechanism is not completely dependable, and a more stable and long lasting system against polyspermic fertilization is required. The oocyte plasma membrane block to polyspermy has been described in several mammalian species such as humans, mice, and rabbits [24-26]. In some invertebrates, a depolarization of the plasma membrane potential prevents sperm from fusing with the oocytes immediately after fertilization [27,28]; however, such an electrical fast block at the level of plasma membrane is not present in mammals. Interestingly, a prior study of *in vitro *fertilization of bovine oocytes has suggested that the cumulus oophorus also plays a role in blocking polyspermy by engulfing numerous sperm.

In mammals, the primary mechanism in preventing polyspermic fertilization involves biochemical modifications of the surrounding of fertilized oocytes as well as the gamete itself; depending on the species, polyspermy block resides at the zona pellucida, the oolemma, or the perivitelline space [29,30]. Generally, the establishment of polyspermy block is initiated and achieved by molecules of cortical granule origin following gamete fusion. Cortical granules are membrane bound secretory organelles located in the cortex of unfertilized oocytes in many invertebrates and vertebrates [31-44]. Mammalian cortical granules, which were first described by C.R. Austin in hamster oocytes using phase contrast microscopy, are derived from Golgi complexes during oocyte growth [41,45]. Following fertilization, cortical granules undergo exocytosis to release their contents into the perivitelline space (Figure 1). This secretory process is calcium dependent and is known as the cortical reaction [46-49]. The released cortical granule proteins are responsible for blocking polyspermy by modifying the oocytes' extracellular matrices, such as the zona pellucida in mammals, the vitelline envelope in echinoderms, and the fertilization layers in amphibians [35,42,44,50-55]. Although other biochemical and structural changes in ZP following cortical granule exocytosis are inferred, only the proteolytic cleavage of ZP2 to a form called ZP2f has been experimentally described [56]. This modification of ZP, termed zona reaction or zona hardening, exerts polyspermic block by inhibiting additional sperm to bind ZP and hamper bound sperm to penetrate this ECM [57]. Furthermore, the accumulation of cortical granule components on oolemma and PVS, as well as the fusion of cortical granule membrane with oolemma may play roles in the establishment of a plasma membrane and PVS block to polyspermy by hindering gamete interaction or modifying an incoming sperm [29,44].

Mammalian cortical granules range in size from 0.2 um to 0.6 um in diameter and appear morphologically similar to each other at the ultrastructural level [41,58-60]. Cortical granules are distinct and different from most other regulatory secretory organelles in that they are not renewed once released. These granules are only synthesized in female germ cells and transform an egg upon sperm entry; therefore, this unique organelle has inherent interest for our understanding of the fertilization process. The following review will focus on the biology and dynamics of cortical granules in mammals; topics such as the formation, distribution, pre-fertilization release of cortical granules, molecular mechanisms that regulate granule secretion, granule contents (including biological functions if available), as well as granule population heterogeneity, will be discussed.

## Formation, distribution, and pre-fertilization release of cortical granules

Mammalian cortical granules first appear in the early stages of oocyte growth; however, the exact window of time in which cortical granules are synthesized is different from species to species [41]. In rat and mouse, cortical granules are first seen in the unilaminar follicle [46,61-63], whereas in the human, monkey, hamsters, and rabbit, they first appear in multilayered follicles [46,64-68]. During the early stage of follicular growth, Golgi complexes undergo hypertrophy and proliferation, and the formation of cortical granules from Golgi complexes occurs at this stage [41,46,69]. At first, small vesicles are formed from hypertrophied Golgi complexes that migrate toward the subcortical region of the oocytes. These vesicles then coalesce to form mature cortical granules that eventually separate from Golgi complexes [41]. Subcellular structures containing both granules and Golgi complex-like organelles have been observed in growing mouse oocytes [70], and these structures likely represent the sites of cortical granule synthesis. The production of cortical granule in mammalian oocytes is a continuous process, and newly synthesized granules are translocated to the cortex up until the time of ovulation [41,46,61,65,70-75]. Several lines of evidence show that the granule migration is a cytoskeleton-dependent process and microfilaments are required for this cortical translocation in both non-mammalian and mammalian animal models, such as sea urchin, porcine, human, and mouse [76-80]. In mammalian oocytes, the migration of cortical granules is an important step in cytoplasmic maturation and has been used routinely as a criterion in assessing the maturity and organelle organization of developing oocytes [81].

**Figure 2:**
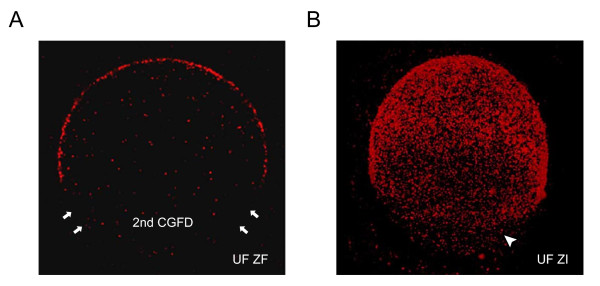
**Confocal scanning laser micrographs of cortical granules in mouse oocytes labeled with lectin LCA**. (A) An equatorial section of unfertilized zona free oocyte and (B) a three-dimensional projection of unfertilized zona intact oocyte showing the cortical granules (in red), the second cortical granule free domain (arrows), and the pre-fertilization release (arrowhead).

**Figure 3:**
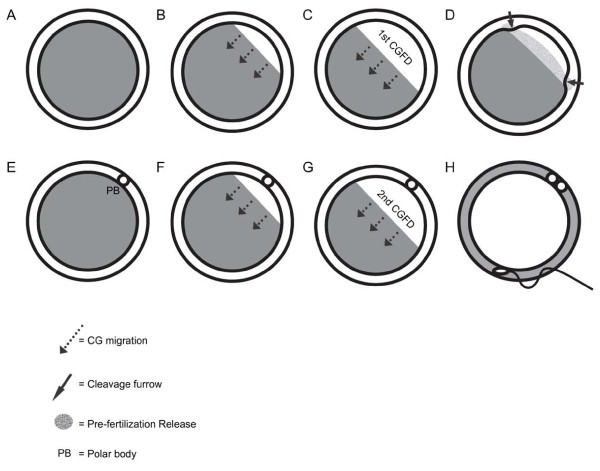
**Formation of cortical granule free domains in mouse oocytes**. Redistribution and pre-fertilization release of cortical granules prior to gamete fusion are shown in this schematic diagram. Unfertilized zona intact oocytes in different developmental stages and fertilized zona intact oocytes are shown chronologically. (A - D) Germinal vesicle intact oocytes (in prophase I) prior to ovulation; (E - G) Metaphase II unfertilized oocytes following ovulation; (H) Fertilized oocyte. Physical localization of cortical granules in the cortex is shown with areas in grey shade. Cortical granules are evenly distributed in the cortex of germinal vesicle intact oocytes prior to ovulation (A). A small population of granules undergoes redistribution (B), and an area devoid of cortical granule (i.e. first CGFD) is formed (C). During polar body extrusion, pre-fertilization release of cortical granules is evident in the cleavage furrow (D). Following ovulation, cortical granules undergoes redistribution (F), and an area devoid of cortical granules (i.e. second CGFD) is formed again (G). Following sperm penetration, the remaining cortical granules undergo exocytosis and release their contents into the extracellular space (H). Arrow with dotted line = cortical granule migration; arrow with solid line = the site of cleavage furrow; mosaic grey shade = cortical granule pre-fertilization; PB = polar body.

Translocated cortical granules are evenly distributed in the cortex of unfertilized oocytes; however, some granules undergo complex changes in distribution prior to ovulation [82-84], in results of areas devoid of cortical granules (Figure 2). This meiotic spindle-associated granule-free area was first noted 30 years ago in mouse oocytes, although the extent of this domain apparently was not fully appreciated [46,71]. Recent studies have reported the development of two distinct cortical granule free domains (CGFDs) over the metaphase I and metaphase II spindles in hamster and mouse oocytes, respectively [60,70,82,84-88]. Interestingly, CGFD appears to be unique to rodents since feline, equine, bovine, porcine, and human oocytes lack such a domain [75,89-93]. Several lines of evidence show that redistribution and/or exocytosis of cortical granules during meiotic maturation are likely mechanisms for the formation of CGFDs, supported by the observations of an increase in granule density in the periphery of the CGFD and a decrease in the total number of cortical granules during meiotic maturation [82,83,85]. In mice, redistribution of granules appear to be the main factor contributing to development of the first CGFD over metaphase I spindle since cortical granule exocytosis is not evident this time [88] and the formation of this domain is not inhibited in the presence of BAPTA, a Ca^2+ ^chelator that prevents cortical granule exocytosis [87]. However, a study on hamster oocytes reported that small extend of cortical granule release was observed during metaphase I, and both granule migration and exocytosis are claimed to be required for generating first CGFD [82]. The exact mechanisms responsible for the first CGFD formation in rodents may be different from species to species and will need to be determined experimentally. The second CGFD formed over the spindle in metaphase II oocytes is thought largely due to redistribution of cortical granules, supported by the observation of increased granule density at the edge of the granule-containing domain [87,88]. Studies in mice also reveal that this cortical granule migration during metaphase I and II transition is chromatin-mediated [87]; however, disruption of the spindle with microtubule inhibitors does not prevent formation of a CGFD over the chromatin [80]. While the formation of both cortical granule free domains appears to occur by granule redistribution, exocytosis has been detected during the metaphase I to metaphase II transition at both light and electron microscopy levels [82,86,88]. Interestingly, the pre-fertilization exocytotic event documented during this meiotic transition period does not participate in the second CGFD development and only contributes to the enlargement of the granule-free area since calcium chelator does not abolish the domain formation [87]. Taken together, present findings demonstrate that granule redistribution is the primary mechanism responsible for cortical granule free domain formation in mammals. Changes in cortical granule distribution and formation of CGFDs in mouse oocytes are summarized in Figure 3. Prior study of mouse oocytes reveals that the pre-fertilization exocytosis of cortical granules during metaphase I and II transition involves a specific population of granules [88]. In addition, this exocytotic event takes place during polar body extrusion and only in the cleavage furrow. The finding is supported by the observation that the mean total numbers of cortical granules per oocyte decreases after polar body extrusion (from ~7400 to 4100) [85]. Moreover, an electron microscopic study of cortical granules revealed that granules undergoing exocytosis prior to gamete fusion were predominantly confined to an ultrastructurally "light" population [60]. Aside to the pre-fertilization release aforementioned, small populations of cortical granules have been described to undergo exocytosis at different time prior to fertilization. One wave of cortical granules release occurs during metaphase I and has been reported only once in thin sections of hamster oocytes [82]. Another wave of the release, which has only been observed ultrastructurally, takes place after addition of sperm but before sperm penetration when oocytes are inseminated *in vitro *[60]. However, comparable exocytosis of granules in other mammals has not been described.

Based on the preceding review, we know that pre-fertilization exocytosis of cortical granules, as well as formation of cortical granule free domains, that take place at a specific time and place are well documented in some mammals. Although the exact biological significance and functions of CGFDs and pre-fertilization release of cortical granules are elusive, several hypotheses are available. First, the formation of CGFDs ensures that the first and second polar bodies to be extruded with few cortical granules in them; therefore allowing maximum number of cortical granules to exert their biological function(s) at the right window of time. Second, the pre-fertilization release of cortical granules during the metaphase I and II transition may be involved in modification of zona pellucida, perivitelline space, and/or oolemma directly above the site of the release, thereby minimizing the likelihood for sperm to bind and penetrate oocytes through this area where maternal chromatin is located. Establishment of this local block to sperm entry will reduce the possibility of damaging maternal genetic materials when paternal chromatin undergoes decondensation following gamete fusion. This may be particular importance in rodents in which the oocyte surface area is typically 25% that of larger mammals and therefore the risk is much higher for sperm entry in this region. This hypothesis is supported by findings showing that sperm-oocyte fusion occurs less frequently in the CGFD [60,94]. Lastly, the limited cortical granule exocytosis occurs during oocyte maturation and prior to fertilization may condition the zona by altering the physico-chemical properties slightly in such a way that the zona can be penetrated only by a very "strong" sperm [82,86]. This postulation is supported by finding from an early report on the loss of cortical granules and the development of CGFD in oocytes matured *in vitro *cultures are accompanied by small degree of ZP2 to ZP2f conversion [83]. In conclusion, most but not all mammalian cortical granules under exocytosis following gamete membrane fusion [41]. Despite the general function of cortical granules in establishing block to polyspermy, it is highly probable that CGFDs and pre-fertilization release perform unique biological function(s) since they occur in a specific location at a specific time. However, the exact physiological functioning of these events still needs to be determined experimentally.

## Regulatory mechanisms of cortical granule docking and exocytosis

### Soluble NSF-attachment protein receptors (SNARE proteins)

**Figure 4:**
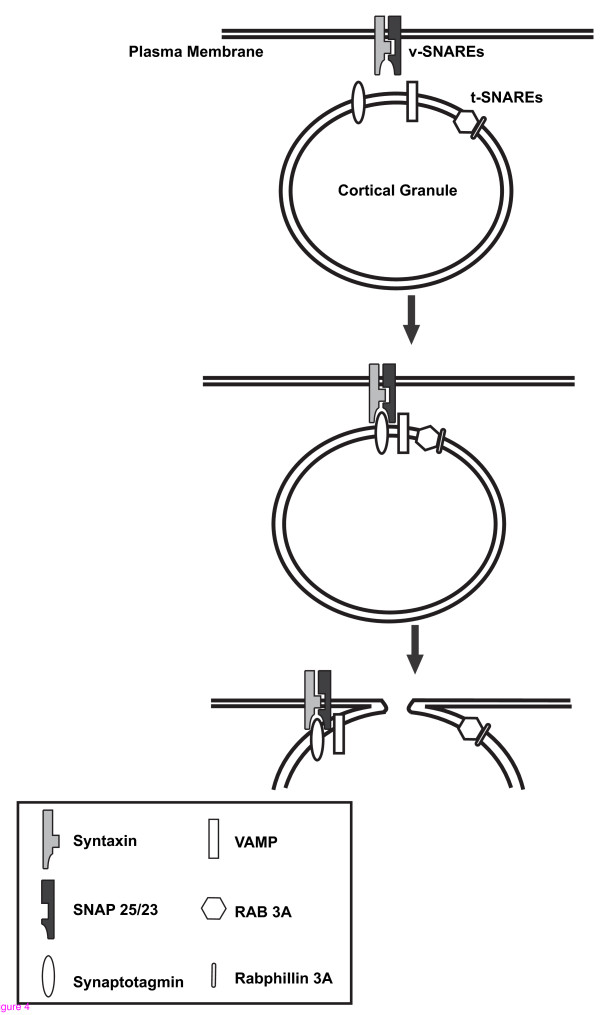
**Representation of interaction between SNARE proteins dictating the exocytosis of cortical granules**. Vesicle-associated v-SNAREs (VAMP and synaptotagmin) interact with cognate target membrane-associate t-SNAREs (Syntaxin and SNAP-25/23) and the recognitions of these molecules govern vesicular trafficking and membrane fusion in cortical granule reaction. In addition to the SNARE proteins, Rab 3A and Rabphilin3A, are also involved in regulating membrane fusion.

Recently, two classes of proteins, known as v- and t-SNAREs (soluble NSF-attachment protein receptors), have been demonstrated to play important roles in mediating vesicle docking and membrane fusion. Members of v-SNARE, such as VAMP (vesicle- associated membrane protein) and synaptotagmin, are found on vesicle membranes, while members of t-SNARE, such as syntaxin and SNAP-25 (synaptosome-associated protein of 25 kDa), are found on target membranes [95,96]. Prior to vesicle exocytosis, the v-SNAREs on vesicle membranes, along with another small GTP-binding protein, Rab, bind to appropriate t-SNAREs on target membranes, resulting in docking of vesicles to target membranes. The binding of v-SNAREs and t-SNAREs also leads to the formation of a stable protein complex that is necessary for inducing membrane fusion [95-100]. Convincing evidence of interactions between SNARE proteins dictating the exocytosis of cortical granules is presented in several mammalian species. In mice, unfertilized oocytes have been demonstrated to contain the t-SNARE protein SNAP-25, which is essential for the cortical reaction since the presence of botulinum neurotoxin A selectively cleaves SNAP-25 and inhibits sperm-induced cortical granule exocytosis [101]. A recent work in porcine oocytes reveals that t-SNARE protein SNAP-23 and v-SNARE protein VAMP1 are involved in docking cortical granules to the oolemma, and interactions between these SNARE proteins, as well as with the molecule complexin, are responsible for arresting cortical granules in the cortex of the oocytes prior to exocytosis [16]. Additionally, Rab3A, a member of small GTP-binding Rab family, is present in mouse oocytes with a cortical localization in unfertilized oocytes [102]; however, the involvement of Rab3A in cortical granule exocytosis has not been examined. A putative target protein of Rab3A, Rabphilin-3A, is expressed in mouse oocytes, and microinjection of N- and C-terminal fragment of recombinant Rabphilin 3A inhibits cortical granule exocytosis in a dose-dependent manner, demonstrating that Rabphilin 3A plays a role in mediating cortical reaction in mammalian oocytes [103]. Interaction between SNARE proteins dictating the exocytosis of cortical granules is summarized in Figure 4. Taken together, these observations demonstrate that exocytosis of cortical granules in mammalian oocytes is regulated by a SNARE protein-mediated pathway.

### Calcium-dependent pathways

During oogenesis, cortical granules are translocated to the cortex of unfertilized oocytes and remain positioned several microns below the plasma membrane of the oocytes until stimulated to exocytose. The fusion of sperm plasma membrane with the oolemma, which subsequently activates a series of intracellular signaling pathways, is known to be responsible for the occurrence of cortical reaction [48]. Since calcium ionophore induces cortical granules exocytosis [75,85,104] and the calcium chelator, BAPTA, inhibits this process [47]; elevation of cytosolic calcium is thought to be required for this exocytotic event to take place [46-49]. The complete signal-transducing pathway accountable for cortical granule exocytosis is not yet completely understood. Nevertheless, several studies have revealed that the G proteins appear to mediate the signal transducing cascade leading to the release of cortical granule contents in mammalian oocytes [105-108]. This conclusion is supported by the observation that microinjection of GTP-α-S, a G protein activator, induces cortical reaction in hamsters, sheep, and pig oocytes [105,107]. Furthermore, a G protein-mediated PIP_2 _cascade that involves the IP_3 _production has been implicated in calcium-dependent cortical granule exocytosis. In mammals, a sperm-specific phospholipase C zeta is introduced into the oocyte cytoplasm upon fertilization and participates in the generation of IP_3 _[109,110]. In a study of hamster oocytes, microinjection of IP_3 _causes intracellular calcium release and induces cortical granule to undergo exocytosis [105,106]. Similarly, ZP modification, which is a result of cortical reaction, was reported when IP_3 _was microinjected into metaphase II mouse oocytes, indicating that IP_3 _plays a role in a signal transducing cascade that mediates exocytosis of cortical granule [111,112]. Lastly, microinjection of monoclonal antibody 18A10, which inhibited IP_3_-induced calcium release by binding to the IP_3 _receptor, inhibited ZP2 to ZP2f conversion in a dose-dependent manner, indirectly demonstrating inhibition of the cortical reaction [113]. Taken together, these observations support that idea that IP_3 _is involved in mediating cortical granule exocytosis in mammalian oocytes.

A large body of evidence shows that the other pathway of the G protein-mediated phosphatidylinositol biphosphate (PIP_2_) cascade, protein kinase C (PKC) activation by diacylglycerol (DAG), may also function in regulating cortical reaction in mammalian oocytes. A recent study in rats demonstrates that the receptors for activated PKC shuttle activated PKC to the oocyte cortex in order to facilitate cortical reaction [114]. In addition, studies in rat, porcine, mouse, and human oocytes showed that cortical granules were induced to undergo exocytosis when oocytes were treated with PKC activators DAG or phorbol ester [79,112,115-118]. However, the cortical granule exocytosis induced by phorbol esters does not faithfully mimic that seen in fertilized oocytes, and the presence of PKC inhibitors did not result in detectable inhibition of granule release. These findings suggest that PKC alone is not sufficient to stimulate cortical granule to exocytose and the participation of inositol trisphosphate (IP_3_), as well as intracellular free calcium, appears to be necessary in regulating this biological process. Finally, injection of Ca2+/calmodulin-dependent protein kinase (CaMKII) cDNA induces moderate extent of cortical granule exocytosis in mouse oocyte, suggesting CaMKII is also involved in but is not alone capable of inducing cortical reaction. In conclusion, cortical reaction in mammalian oocytes is calcium-dependent, and this exocytotic event is mediated by G protein signal-transducing cascade, in which IP_3 _induces the increase of intracellular calcium. Modulating molecules such as PKC and CaMKII also function in initiating this secretory process.

## Cortical granule contents

The total number of mammalian cortical granule proteins is not known; however, it has been estimated to be between four and fourteen [56,119,120]. The amount of cortical granule material available in mammalian oocytes (estimated to be picogram quantities/oocyte) is scanty, which is a major factor that hampers studies on granule constituents. The lack of specific probes to mammalian cortical granules makes the characterization of cortical granules even more difficult. Nevertheless, several molecules have been inferred or demonstrated to be of cortical granule origin. Components identified and characterized in mammalian cortical granules will be described in the following section.

### Glycosylated components

Several studies using polysaccharides-binding dyes or probes reveal that mammalian cortical granules are rich in carbohydrates [29,46,67,121-128]. The first cytochemical demonstration of mammalian cortical granules was done using periodic acid Schiff's reagent (PAS), which recognizes polysaccharides [126], to stain paraffin embedded sections of unfertilized hamster oocytes [128]. No cortical granules were detected in the cortex of the fertilized oocytes; however, PAS positive materials, presumably of cortical granule origin, were abundant in the perivitelline space soon after the sperm penetration. Subsequently, hamster and rabbit cortical granules were labeled ultrastructurally with periodic acid with thiocarbohydrazide-OsO_4_/silver protein, and phosphotungstic acid, which both bind to sugar residues [67,127]. Ruthenium red, a polycationic inorganic dye that can be used in electron microscopy, has also been used to label negatively charged substances such as glycoproteins [123]. The disadvantage of ruthenium red for biological staining is that the dye is not membrane permeable; however, it is useful for stabilizing and demonstrating glycosylated extracellular materials in ultrastructural studies [29,46,121,122]. Ultrastructural work on *in vivo *fertilized rabbit oocytes revealed round masses of intensively stained ruthenium red material that resembled exocytosed cortical granules in crypt-like invaginations of the oolemma [122]. The ruthenium red labeled materials were interpreted to be exocytosed cortical granule contents since they resembled recently released cortical granules and were detected on the surface of fertilized oocytes. An electron microscopic study on *in vivo *fertilized rat and hamster oocytes also showed ruthenium red stained material freshly exocytosed from the cortical granules on the oolemma and in the perivitelline space of the oocytes [46]. Colloidal iron has also been used to localize negatively charged residues such as carbohydrates in electron microscopic studies [124]. *In vivo *fertilized rabbit oocytes stained with colloidal iron had more negatively charged residues on the oolemma than unfertilized oocytes [125]. This increase in oolemmal staining was interpreted to be due to the release of the cortical granule contents following sperm penetration. Taken together, these observations indicate that mammalian cortical granules contain and are enriched with glycosylated materials.

Lectins are proteins (often of plant origin) that recognize and bind to carbohydrate moieties of glycoconjugates. To date, more than a dozen of lectins have been used cytochemically to demonstrate cortical granules in unfertilized oocytes and/or to localize cortical granule exudates following fertilization [43,88,93,129]. The lectins *Lens culinaris *agglutinin (LCA) and *Canavalia ensiformis *agglutinin (ConA) that are specific for α-D mannose have been used to label the cortical granules of human, mouse, hamster, cat, and equine oocytes [25,58,85,88,89,93,130,131]. In human and mice, LCA binds cortical granules at the light or electron microscopic levels (Figure 2) [25,85,88,131]. Western blots of unfertilized oocytes probed with LCA reveal murine cortical granules contain at least 15 mannosylated proteins [88]. In hamsters, the cortical granules have been shown at both light and electron microscopic levels to bind LCA or ConA [58,130]. Lectin blots of unfertilized oocytes show that at least 11 mannosylated proteins are present in hamster cortical granules and that nine of these glycoconjugates may contribute to the formation of cortical granule envelope [130]. In cats, LCA specifically labels cortical granules at both the light microscopic and ultrastructural levels [89]. The lectin peanut agglutinin (PNA) that is specific for β-D-galactose (especially β-D-galactose (1,3)-D-*N*-acetylgalactosamine residues) recognizes cortical granules in hamster and pig oocytes [130,132]. Following fertilization, PNA-binding cortical granule components are evident on the oolemma of fertilized hamster oocytes and in the perivitelline space of fertilized pig oocytes. PNA blots of unfertilized hamster oocytes show that at least seven cortical granule glycoconjugates contain β-D-galactose and of these six are mannosylated since they are also recognized by ConA [130]. Several lectins such as *Dolichos biflorus *agglutinin (DBA), *Ricinus communis *agglutinin I (RCA_120_), wheat germ agglutinin (WGA), *Datura stramonium *agglutinin (DSA), *Maackia amureusis *agglutinin (MAA), *Aleuria aurantia *agglutinin (AAA), *Helix pomatia *agglutinin (HPA) that are specific for α-D-acetylgalactosamine, galactose, *N*-acetylglucosamine, *N*-acetyllactosamine, *N*-acetylneuraminic acid, fucose, and D-*N*-acetylgalactosamine respectively bind cortical granules in hamsters oocytes at light and electron microscopic levels [129,130]. The lectin WGA and AAA have been shown cytochemically or at the ultrastructural level to label human cortical granules [133,134]. In addition, lectins that are specific to *N*-acetylgalactosamine (*Artocarpus integrifolia*, AIA), N-glycolylneuraminic acid (*Limax flavus *LFA), as well as more complex carbohydrate moieties (*Datura stramonium *DSA, *Maclura pomifera *MPA, and *Phaseolus vulgaris *PHA-E) have been shown to label cortical granules in human oocytes at the electron microscopic level [134]. Finally, the appearance of specific fucosyl and sialyl-rich glycoconjugates, probably of cortical granule origin, in the perivitelline space has been reported in human and mouse oocytes using the lectins *Limulus polyphemus *agglutinin (LPA), *Lotus tetragonolobus *agglutinin (FBP), and *Ulex europaeus *agglutinin (UEA) [135,136]. In conclusion, mammalian cortical granules are rich in carbohydrates, and the carbohydrate moieties on glycosylated constituents are complex.

### Proteinases

Several proteinases have been inferred or demonstrated to be present in mammalian cortical granules [43]. They are thought to be involved in ZP modification and hardening, which result in blocking polyspermy and protecting preimplantation embryos [50,137-139]. The presence of proteinase in mammalian cortical granules was first reported in hamsters [140]. Cortical granule exudate of electrically activated hamster oocytes induced zona modification of unactivated hamster oocytes, which resulted in inhibition of sperm adherence to or penetration through the ZP. A similar inhibitory effect was observed when cortical granule exudate of *in vitro *fertilized mouse oocytes was used [141]. The presence of trypsin inhibitors such as soybean trypsin inhibitors (SBTI) and p-aminobenzamidine (PAB) alleviated this inhibitory effect. Furthermore, when mouse oocytes were activated by calcium ionophore in the presence of a serine protease inhibitor, leupeptin, the block to polyspermy was not established [142]. A study done by Cherr et al. provided cytochemical evidence for the presence of proteinase in mammalian cortical granules [58]. Fluorescent SBTI bound granules in the cortex of unactivated hamster oocytes and less of this cortical granular labeling by SBTI was observed in activated oocytes. The binding of SBTI to the cortical granules was inhibited when aprotinin and benzamidine hydrochloride were used. Ultrastructurally, biotinylated SBTI recognized by gold-avidin was also shown to bind cortical granules. Interestingly, SBTI bound only the decondensed or the exocytosing cortical granules, but not the condensed granules. It is possible that the activation of this trypsin-like proteinase occurs during granule decondensation, which is necessary for the probe binding to the proteinase, or that decondensation of granules is simply necessary to provide physical access for the probe [58]. Taken together, these findings reveal that mammalian cortical granules contain a trypsin-like proteinase and that this cortical granule proteinase functions in blocking polyspermy.

Tissue-type plasminogen activator (tPA), a serine proteinase that converts plasminogen into its active form plasmin, has been inferred to be a mammalian cortical granule component since it is released from oocytes at fertilization or activation when granules undergo exocytosis [143]. The presence of tPA antibody during oocyte activation inhibited the cortical granule-induced zona hardening and block to sperm penetration, suggesting that tPA functions in blocking polyspermy. However, there are observations inconsistent with tPA being a cortical granule protein. First, tPA has not been demonstrated to be present in the cortical granules of unfertilized oocytes at the cytochemical level. Second, primary oocytes do not contain tPA, and the translation of tPA mRNA is only triggered upon resumption of meiotic maturation after most cortical granules have been synthesized [144,145]. These findings suggest that tPA is not a cortical granule protein. Nevertheless, it is possible that tPA is secreted from vesicles that are distinct from cortical granules or other vesicles rather than from conventional cortical granules following fertilization or oocyte activation, as shown in recent studies of mouse oocyte calreticulin [146,147]. The question of whether mammalian cortical granules contain tPA still needs to be addressed experimentally.

Finally, a proteinase, designated ZP2 proteinase, is released from fertilized and activated oocytes [56]. The proteinase is involved in ZP2 proteolysis, which results in the conversion of ZP2 (120 kDa glycoprotein) to ZP2f (90 kDa glycoprotein), and the enzyme is thought to be responsible for blocking polyspermy [56]. High performance liquid chromatography analysis of oocyte exudate indicates that the ZP2 proteinase has a molecular weight of 21 to 34 kDa. The ZP2 proteinase is insensitive to several inhibitors including metallo, carboxyl, sulfhydryl, and serine proteinases inhibitors; however, the ZP2 antibody or the Fab fragment of this antibody inhibits 70% and 50% of the enzyme activity respectively.

### Ovoperoxidase

An ovoperoxidase has been detected in cortical granules of unfertilized mouse oocytes using the 3,3'-diaminobenzidine (DAB) cytochemical staining method [137,148]. The protein was detected on the plasma membrane, in the perivitelline space, and in the zona pellucida following ionophore activation. The ovoperoxidase staining in activated oocytes could be prevented by its inhibitors phenylhydrazine or sodium sulfite, indicating the labeling is specific. Several lines of evidence suggest that ovoperoxidase catalyzes cross-linking of tyrosines in the zona resulting in hardening of zona pellucida [137]. First, ovoperoxidase inhibitors such as phenylhydrazine, sodium sulfite, sodium azide, and glycine ethyl ester and tyrosine analogs all inhibited zona hardening. Second, the presence of exogenous horseradish peroxidase induced partial hardening of the zona in unfertilized oocytes. Although several studies have demonstrated the presence of peroxidase in cortical granules in invertebrates [149-153], these studies done by Schmell and Gulyas are the only reports of peroxidase in mammalian oocytes [137,148].

### Calreticulin

Calreticulin is a chaperone protein in the endoplasmic reticulum or a lectin involved in chaperoning glycoproteins [154-156]. The protein was inferred to be in hamster cortical granules since immunolabeling with anti-calreticulin antibody was located in the cortex of the unfertilized oocytes and appeared to be granular [146]. Oocytes double labeled with the cortical granule specific lectin LCA and calreticulin antibody showed co-localization of the two probes in many granules, indicating the presence of calreticulin in cortical granules. Calreticulin can be detected on western blots of the oocyte's activation medium demonstrating the protein was exocytosed in response to oocyte activation. The authors of this study proposed that calreticulin may function as a chaperone for other exocytosed cortical granule components that function in blocking polyspermy. They also suggested calreticulin acts as a lectin that binds and blocks the carbohydrate moieties of glycoproteins important for oocyte-sperm interaction, thereby blocking polyspermy. However, more experimental data are required to support these postulates. A recent study using mice has demonstrated that calreticulin is released by vesicles in the cortex other than cortical granules since mouse oocytes double labeled with cortical granule specific lectin LCA and calreticulin specific antibody did not show co-localization of two probes [147]. Following its release, calreticulin was shown to be present on the extracellular surface of the oocyte's plasma membrane and in the perivitelline space, and the protein was demonstrated to interact with the cytoskeleton of the oocyte and mediate transmembrane signaling that leads to cell cycle resumption [147]. The discrepancy in findings of two studies and the exact origin of calreticulin need to be resolved experimentally.

### *N*-Acetylglucosaminidase

*N*-acetylglucosaminidase, a glycosidase that has high specificity for terminal *N*-acetylglucosamine residues, was localized in mouse cortical granules at the immunoelectron microscopy level [157]. The enzyme activity was detected in oocytes exudates following artificial activation. The N-acetylglucosaminidase found in mouse cortical granules has maximal activity at pH 4.5 to 5.5 and was identified as the β-hexosaminidase B isoform (β, β homodimer). Oocytes activated in the present of *N*-acetylglucosaminidase inhibitor or antibodies showed a significant increase in the number of sperm binding to the zona when oocytes were fertilized *in vitro *[157]. This result indicates that *N*-acetylglucosaminidase is required for the mouse zona block to polyspermy, which removes the terminal *N*-acetylglucosamine residues on zona pellucida to abolish sperm binding [157,158]. Although *N*-acetylglucosaminidase is also found in the cortical granules of *Xenopus *oocytes, it is not known if this enzyme is present in oocytes of other mammals.

### p32

p32 is a mouse cortical granule component recognized specifically by the monoclonal antibody 3E10, which was made against calcium ionophore-induced oocyte exudates [119], and the protein has a molecular weight of 32 kDa on western blots. In metaphase II oocytes, the 3E10 antibody stained granules in the cortex, and no labeling was observed in the region over the spindle. Oocytes double labeled with 3E10 and LCA, which specifically labels mouse cortical granules [85], exhibited a nearly complete co-localization, demonstrating the presence of the 3E10 antigen in mouse cortical granules. In fertilized oocytes and two-cell embryos, a very small amount of p32 remained on the oocyte or embryo surfaces. The presence of p32 could not be detected in eight-cell embryos, morulae, or blastocysts. p32 appears not to play a role in blocking polyspermy since the 3E10 antibody did not interfere with the ability of fertilized oocytes to establish such block. Interestingly, 3E10 staining was significantly reduced or almost undetectable in fertilized oocytes. This observation suggests that the released p32 is only functionally active during the early period of fertilization or the protein undergoes conformational change following its release from the cortical granules so that the 3E10 epitope is no longer available.

### Peptidylarginine deiminase (PAD/ABL_2 _antigen/p75)

p75 is a mouse cortical granule component recognized specifically by the polyclonal antibody ABL_2_, which was made against zona free whole mouse blastocysts [120]. The protein has a molecular weight of 75 kDa, and two-dimensional gel electrophoresis of mouse oocytes revealed that p75 exists as four isoforms with isoelectric points between 4.9 and 5.3. The protein was localized to mouse cortical granules at the immunoelectron microscopy level, and it was released following *in vitro *fertilization [120]. The synthesis of p75 was first detected in < 20 μm oocytes and continued to increase to about 1.5% of total protein synthesis during oocyte growth [159]. In metaphase II oocytes, p75 synthesis decreased about 10-fold. Synthesis of p75 was observed following *in vitro *translation of germinal vesicle intact oocyte poly(A)^+ ^RNA, but not of metaphase II oocyte poly(A)^+ ^RNA indicating that p75 synthesis is under translational regulation during oocyte growth and maturation. To identify this cortical granule protein, Liu et al. immuneprecipitated p75 from mouse ovarian lysate and the protein was sequence by tandem mass spectrometry. Based on a partial amino acid sequence, p75 was identified to be peptidylarginine deiminase (PAD) [160]. This study is the first report on biochemical identification of mammalian cortical granule component based on protein sequence. Interestingly, PAD appears to be a non-glycosylated secretory protein and plays a regulatory role after its release at fertilization in embryonic cleavage and early preimplantation development. In a study of hamster cortical granules, the ABL_2 _antibody was shown to recognize two cortical granule proteins designated p62 and p56, which have molecular weights on western blots of 62 and 56 kDa, respectively [161]. Following fertilization, both hamster cortical granule proteins were released and contributed to the formation of the cortical granule envelope, an extracellular matrix in the perivitelline space of fertilized mammalian oocytes following cortical reaction [121,161,162]. An *in vivo *functional study showed that p62 and p56 did not appear to be involved in blocking polyspermy since fertilization remained monospermic in all oocytes when treated *in vivo *with the ABL_2 _antibody. Furthermore, *in vivo*treatment of 2-cell hamster embryos with the antibody inhibited cleavage and delayed blastocyst formation in a dose-dependent manner, in consist with the observation that the mouse PAD of cortical granule origin functions in preimplantation development. Interestingly, cortical granule proteins, p62 and p56, in hamster appeared to be immunologically related to the sea urchin cortical granule protein hyalin since they were recognized by the antibody IL2 that was made against purified *S. purpuratus *hyalin [163]. The conclusion is further supported by the finding that IL2 antibody inhibited hamster 2-cell embryos cleavage *in vivo*. However, the exact molecular identities of these ABL_2_-binidng cortical granule proteins in hamster oocytes still need to be determined. Taken together, PAD is present in mouse cortical granules and plays a regulatory role in preimplantation development. It will be interesting to examine whether PAD is also present in oocytes of other rodent species and the antibody ABL_2 _reacts and recognizes proteins of cortical granule origin in other mammals.

## Cortical granule populations

Different types of cortical granules have been described in unfertilized oocytes of some species such as fish, amphibians, crustaceans, echinoderms, and mammals [34,37-40,58,60,74,164-167]. In the next section, works focused on granule population in non-mammalian species will be presented first, followed by studies in mammals, to provide a more fundamental understanding and broader perspective on cortical granule heterogeneity.

In the Zebrafish, *Brachydanio rerio*, two types of cortical granules, the dark type (electron-dense) and the light type (electron-lucent), have been identified at the electron microscopy level [38]. Differential cytochemical staining with either Alcian blue or toluidine blue also confirms the presence of these two types of cortical granules in unfertilized oocytes of Zebrafish [38]. The killifish, *Fundulus heteroclitus*, has two populations of cortical granules; at the ultrastructural level, one population contains filamentous and/or granular material, while the other does not [39]. In the frog, *Xenopus laevis*, two types of cortical granules are differentially distributed in unfertilized oocytes [34]. One type has a homogeneous matrix of moderate electron density and is located in the animal hemisphere, while the other type has a loose flocculent material and is located in the vegetal hemisphere [34]. In the horseshoe crab, *Limulus polyphemus *L., two types of cortical granules have been described, one appears to be electron-translucent and the other to be electron-dense [37]. In lobsters, *Homarus americanus *and *H gammarus*, four types of cortical granules, that are distinguishable based on their ultrastructural appearance, are present in the cortex of ovarian oocytes and are referred to as high-density, low-density, moderately dense, and ring cortical granules. The contents of each type of granule are released in sequence from the cortex of the oocyte into the perivitelline space following fertilization [40]. In the sea urchin, *Strongylocentrotus purpuratus*, the presence of biochemically distinct populations of cortical granules has been shown based on the immunocytochemical observations. One population of cortical granules contains an antigen recognized by two specific antibodies, 1G8 and B2C2, while the other population does not [164]. In another species of the sea urchin, *Lytechinus variagatus*, four types of granules have been identified immunocytochemically in the unfertilized oocytes [165]. These granules ultimately migrate to the cortex and sequentially release their contents into the perivitelline space upon fertilization.

Mammalian cortical granules are generally similar in morphology [46,58,67,168,169] although dark and light granules have been reported based on their ultrastructural appearance [58,60,74,166,167]. Unlike some species such as fish, amphibians, crustaceans, echinoderms, heterogeneity of cortical granules in mammals is well documented but poorly understood. In humans, two populations of cortical granules of which one has a homogeneous electron-dense core and the other has granular or fluffy contents are present in unfertilized oocytes [166,167]. In pig, hamster, and mouse oocytes, dark and light forms of cortical granules have also been described based on their electron density [58,60,74]. It is not known if this difference in electron density represents different stages in granule maturation, different types of granules, or different stages in exocytosis [58,60,170]. Not till recently, biochemically distinct populations of cortical granules are demonstrated cytochemically in unfertilized mouse oocytes. By taking advantage of two probes specific to mammalian cortical granules, lectin LCA (mannosyl glycoconjugate-binding) and the ABL_2 _antibody (PAD protein-binding), two populations of cortical granules, of which one contains PAD and the other lacks of this molecule, are shown to be present in unfertilized oocytes [88]. However, whether the heterogeneity in granule populations corresponds to different biological functioning is still a question needs to be answered.

## Concluding remarks

To date, only several partially characterized molecules have been reported to be of cortical granule origin in mammals. Cortical granules of invertebrates (e.g. sea urchins and starfish) and some vertebrates (e.g. fish and frogs) are better studied as these model animals served rich source for experimentation, due to the ease in oocyte isolation and manipulation. Our understanding of mammalian cortical granule biochemistry and biogenesis has been hampered for several reasons. First, characterization of granule components is a laborious task due to the paucity of mammalian cortical granule material available per oocyte and low number of oocytes retrievable per animal. It is estimated that each oocyte (i.e. in mice) contains ~20 ng of total proteins and only 2.5% to 5% of these proteins are from cortical granules [119]. Second, the lack of specific probes for mammalian cortical granules makes analytical tests difficult to perform. Studies of cortical granules in humans can be even more troublesome since experimental samples are mostly obtained from volunteer donors, who undergo reproductive assistant treatments, and the number and quality of oocytes collected cannot always be optimal. Although non-mammalian animals serve as excellent models and provide research basis for mammalian species, one should keep in mind that direct extrapolation of information between mammalian and other animal modes requires caution because cellular and molecular differences among different species can affect the experimental outcome.

From the preceding review, we know that mammalian cortical granules are secretory organelles, which are stimulated to undergo calcium dependent and SNARE protein-mediated exocytosis upon gamete fusion, in result of a block to polyspermy. Evolved and extended from the historical viewpoint, mammalian cortical granules are more complex and have more functions than previously realized. Biochemical heterogeneity and complex redistribution of granule in the cortex of unfertilized oocytes prior to ovulation are documented. A small population of granules is evident to exocytose prior to sperm penetration; a number of granule components are reported to function following fertilization in regulating embryonic cleavage and preimplantation development. In addition to providing biological basis to better understand reproduction process, new findings of mammalian cortical granules have made scientists to start apprehending their clinical relevance. Biological parameters of cortical granules, such as granule synthesis and translation to the cortex, are used routinely as criterion to determine the developmental stage of human oocyte grown *in vivo *or *in vitro*. Timely release of these granules is essential for a successful fertilization to occur, especially for individuals who are reproductively challenged and require artificial assistance. Interestingly, low fertilization rate was observed following cryopreservation of mouse oocytes and this low rate was attributed to cryopreservant-induced premature cortical granule discharge [171,172]. Previous study of the cortical granules in unfertilized human oocytes from failed IVF trials also showed a significant decrease in number of granules, suggesting that premature release of cortical granules and early establishment of block to polyspermy prior to insemination may be the cause for some unsuccessful cases of *in vitro *fertilization [173].

Cortical granules are first identified in sea urchins by Harvey in 1910 [174], inspired by Derbès' observation of the fertilization envelope 150 years ago. Not till four decades later, mammalian cortical granules were documented in hamster oocytes by Austin [45]. Fifty five years of time is full of excitement as we have witnessed many technological breakthroughs in reproductive biology and fascinating scientific discoveries of mammalian cortical granules. However, this unique organelle is far from being fully appreciated and a broader perspective on its biochemistry and functional role(s) should be developed and presented. On the quest to find answers to these questions we encounter in studies of mammalian cortical granules, continuous efforts made by scientists in the field of reproductive biology should led us a step closer to better understanding the nature of cortical granules and embracing the beauty of this organelle.

## List of abbreviations used

AAA: *Aleuria aurantia *agglutinin; AIA: *Artocarpus integrifolia*; CaMKII: Ca2+/calmodulin-dependent protein kinase; CGFDs: cortical granule free domains; ConA: *Canavalia ensiformis *agglutinin; DAB: 3,3'-diaminobenzidine; DAG: diacylglycerol; DBA: *Dolichos biflorus *agglutinin; DSA: *Datura stramonium*; DSA: *Datura stramonium *agglutinin (DSA); ECM: extracellular matrix; FBP: *Lotus tetragonolobus agglutinin*; HPA: *Helix pomatia *agglutinin; IP_3_: Inositol trisphosphate; LCA: *Lens culinaris *agglutinin; LFA: *Limax flavus*; LPA: *Limulus polyphemus *agglutinin; MAA: *Maackia amureusis *agglutinin; MPA: *Maclura pomifer*; PAB: p-aminobenzamidine; PAD: peptidylarginine deiminase; PAS: periodic acid Schiff's reagent; PHA-E: *Phaseolus vulgaris*; PIP_2: _phosphatidylinositol biphosphate; PKC: protein kinase C; PNA: peanut agglutinin; PVS: perivitelline space; RCA_120_*: Ricinus communis *agglutinin I; SBTI: soybean trypsin inhibitors; SNAP: synaptosome-associated protein; SNARE: soluble NSF-attachment protein receptors; tPA: tissue-type plasminogen activator; UEA: *Ulex europaeus *agglutinin; VAMP: vesicle- associated membrane protein; WGA: wheat germ agglutinin; ZP: zona pellucida.

## Competing interests

The authors declare that they have no competing interests.
